# The Adaptive Significance of Sensory Bias in a Foraging Context: Floral Colour Preferences in the Bumblebee *Bombus terrestris*

**DOI:** 10.1371/journal.pone.0000556

**Published:** 2007-06-20

**Authors:** Nigel E. Raine, Lars Chittka

**Affiliations:** School of Biological and Chemical Sciences, Queen Mary, University of London, London, United Kingdom; University of Bristol, United Kingdom

## Abstract

Innate sensory biases could play an important role in helping naïve animals to find food. As inexperienced bees are known to have strong innate colour biases we investigated whether bumblebee (*Bombus terrestris*) colonies with stronger biases for the most rewarding flower colour (violet) foraged more successfully in their local flora. To test the adaptive significance of variation in innate colour bias, we compared the performance of colour-naïve bees, from nine bumblebee colonies raised from local wild-caught queens, in a laboratory colour bias paradigm using violet (bee UV-blue) and blue (bee blue) artificial flowers. The foraging performance of the same colonies was assessed under field conditions. Colonies with a stronger innate bias for violet over blue flowers in the laboratory harvested more nectar per unit time under field conditions. In fact, the colony with the strongest bias for violet (over blue) brought in 41% more nectar than the colony with the least strong bias. As violet flowers in the local area produce more nectar than blue flowers (the next most rewarding flower colour), these data are consistent with the hypothesis that local variation in flower traits could drive selection for innate colour biases.

## Introduction

Animals are constantly exposed to stimuli differing widely in their potential importance. Modulation of these stimuli by the sensory systems and cognitive processes allow the animal to assess their relative salience and select appropriate behavioural responses to the most important. One mechanism through which such adaptive behavioural outcomes are promoted is through sensory biases, either within the sensory system or subsequent cognitive processes, causing animals to respond more strongly to certain, more pertinent, stimuli [1], [2]. Although sensory biases have received attention in the context of animal signalling, predominantly relating to mate choice [3], [4] and predator avoidance [5], the potential adaptive role of such biases has not been studied in a foraging context where they could also be very influential [6]. The flower choices of pollinators represent a good model system in which to study the adaptive role of sensory bias in the context of foraging. Flowers send out signals to attract the attention of potential pollinators in a competitive market place, and pollinators are attuned to particular traits, such as the colour, morphology, scent and temperature of the flowers they visit to find food [7]–[10].

Naïve animals must initially use innate rules to find food. Pollinators, such as bees, might use colour as a way to find flowers when first exploring the world [11], [12]. Sensory biases towards particular colours might help naïve bees find flowers, and perhaps even help them to locate the most profitable ones in the local area. Indeed, newly emerged bees, that have never seen flowers, show distinct sensory biases for certain colours [13]–[15]. The bumblebee *Bombus terrestris* L. shows a strong bias towards violet and blue throughout its geographic range [9], [16], [17]. We hypothesize that these innate sensory biases reflect the colour traits of the most profitable flowers species.

Different flower colours appear to be linked to both the reliability of finding high nectar rewards [18], and average amount of sugar produced by particular flower species [9], [17]. In the local flora for this study, violet flowers were more productive than blue flowers [17] (the next most productive flower colour). If local floral traits do drive selection for local bee colour biases, we hypothesized that bees with a stronger sensory bias for violet (over blue) flowers should forage more effectively in this environment. As social insects, bumblebee reproduction is restricted to a subset of individuals within each colony. Hence for bumblebees, intercolony (rather than inter-individual) trait variation allows us to test the adaptive benefits of sensory bias variation when foraging in the local environment. Since bumblebee colonies produce males and new queens in proportion to the amount of food available to them [19]–[21], we can use colony foraging performance as a robust measure of colony fitness.

Our approach explores intercolony variation of floral colour bias, a heritable foraging related trait [22], within a natural population to measure the extent to which such sensory biases can be regarded as adaptive, i.e. improving the colony foraging performance in their natural environment. We do this by comparing the performance of nine bumblebee colonies in colour bias tests under laboratory conditions with the foraging performance of the same colonies under natural conditions. Using this approach allows us to directly correlate trait variation in sensory bias with a proxy measure of colony fitness (foraging performance).

## Methods

### Laboratory colour bias tests

**Figure 1:**
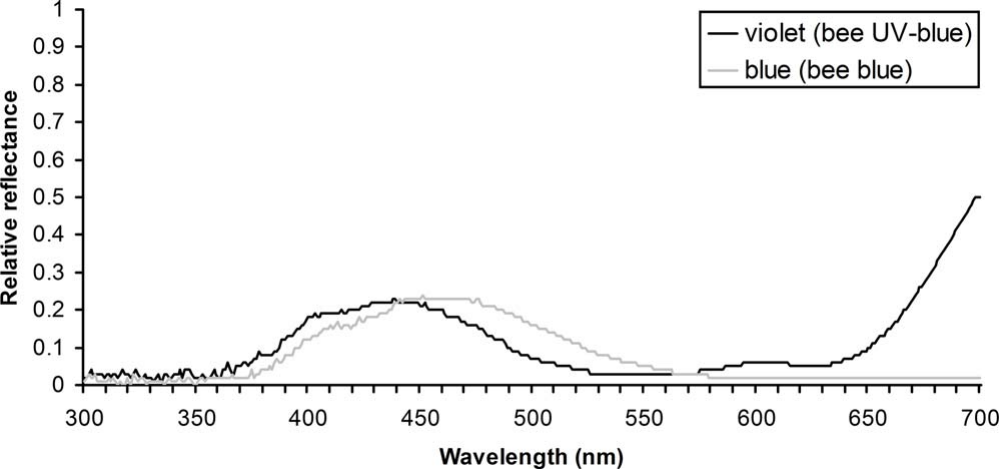
Spectral reflectance profiles of the violet (bee UV-blue) and blue (bee blue) artificial flowers used in colour preference tests. Reflectance can vary from 0 (no reflectance) to 1 (all incident light is reflected). Reflectance functions for each flower type were measured in 1 nm increments over the wavelength range from 300 to 700 nm using a spectrophometer (Ocean Optics S2000) with a deuterium/ halogen light source. Violet (bee UV-blue) and blue (bee blue) flowers differ in the position of their short wave reflectance peak, which are at wavelengths of 435 and 460 nm respectively. Differences in reflectance above 650 nm are not relevant for bees since their visual spectrum ends around that value [46], [47].

**Figure 2:**
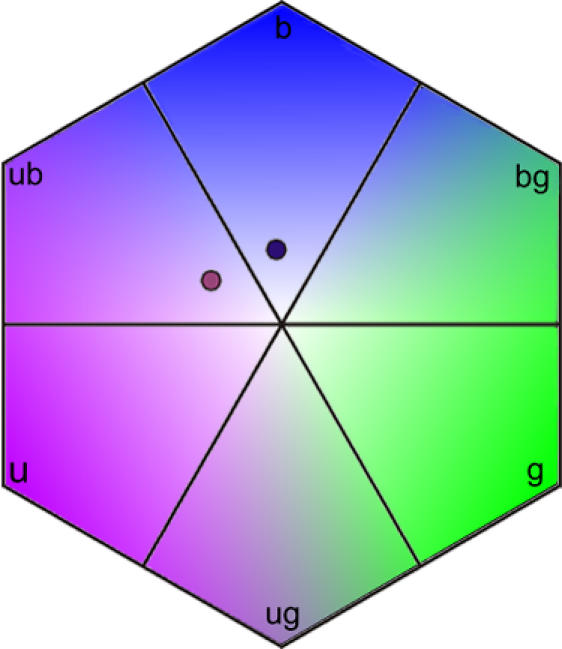
Bee colour hexagon with colour loci of the two flower colours tested. The point generated by a coloured object within the hexagon informs us how bees will perceive the object through their ultraviolet, blue and green photoreceptors, and through further processing of receptor signals in the central nervous system. Each object, such as a flower, is categorised into one of the six bee-subjective colour categories defined by the colour hexagon (ultraviolet (u), UV-blue (ub), blue (b), blue-green (bg), green (g), and UV-green (ug)), depending on which of the three colour receptors of bees (UV, blue or green) they stimulated most strongly [28], [46]. Hence, colours are categorised as bee-blue if they stimulate the bees' blue receptors substantially more strongly than the UV and green receptors, and are categorised as UV-blue if they stimulate the UV and blue receptors more or less equally strongly, but stimulate the green receptor very little, etc. The spectral reflectance of the violet (bee UV-blue) and blue (bee blue) artificial flowers colours was quantified for the spectral properties of the fluorescent lighting used in laboratory colour tests (Fig. 1), and converted into colour loci in bee colour space [28], [48]. These bee-subjective colour loci for the two artificial flower colours used in the laboratory preference tests are indicated by circles coloured as they would appear to humans. The distance between loci for these flower colours is approximately 0.3 colour hexagon units: distances of 0.2 and above are considered easily distinguishable for bumblebees [12], [49].

We tested the innate colour biases of bumblebees (*Bombus terrestris terrestris* L.) by presenting them with artificial flowers in a laboratory flight arena. The nine bumblebee colonies used in this study were raised in the laboratory from nest searching queens caught around Würzburg, Germany. The queens, and subsequent developing colonies, were kept in darkness (except during necessary observations made under dim red light), under controlled temperature and humidity conditions (27°C and 60% relative humidity), and fed pollen-honey paste *ad libitum* prior to experiments. This rearing procedure minimises the risk that intercolony differences are caused by non-genetic factors. As all queens were collected and set up within a few days of each other, this also minimised any intercolony differences in colony age or development when tested. Workers were not exposed to flower colours prior to experiments – hence they began colour bias tests entirely colour-naïve. Nest boxes were connected to a flight arena (120 cm×100 cm×35 cm) in which workers were allowed to forage for 50% sucrose solution (w/w) from 16 colourless, artificial flowers (UV-transmittent Plexiglas plastic squares: 25 mm×25 mm). These colourless, rewarding training flowers were placed on vertical transparent glass cylinders (diameter = 10 mm; height = 40 mm), arranged randomly on the flight arena floor. The spatial positions of these training flowers were regularly reshuffled so that bees would not learn to associate particular arena locations with reward. The sucrose solution reward on these colourless training flowers was presented to the bees in a recessed well in the centre of each flower, and was replenished using a micropipette as soon as it was consumed. All workers in each colony were uniquely identified with individually numbered tags (*Opalith Plättchen,* Christian Graze KG, Germany). We observed the number of foraging trips (bouts) made into the flight arena by each bee to ensure we only tested strongly motivated foragers. For colour preference tests, the 16 colourless, rewarding training flowers were replaced by 16 unrewarding, coloured test flowers: 8 violet (bee UV-blue) and 8 blue (bee blue) targets. The colour bias of each forager was tested individually during a single foraging bout in the flight arena containing the array of coloured, but unrewarding, test flowers. All flowers were changed between foraging bouts to ensure that subsequent test bees received no odour cues from previously tested foragers. Colony colour biases (n = 9 colonies) were calculated by averaging across the 10–15 forager bees tested per colony (101 bees were tested in total). The number of flower choices evaluated per forager ranged from 6 to 58 (mean±1 SE = 16.4±0.9), depending on how long each bee continued to choose unrewarded flowers (1652 flower choices were recorded in total). The violet (bee UV-blue) and blue (bee blue) flowers used in the colour preference tests are easily distinguishable by bumblebees (Figs. 1 and 2, [23]). All training bouts and colour bias tests were performed under high frequency illumination to simulate natural daylight above the bee flicker fusion frequency. Illumination was provided by two ceiling mounted fluorescent lighting rigs, each containing seven light tubes: six DURO-TEST 40W True-Lite tubes and one OSRAM 36W Blacklight tube. The flicker frequency of each strip light was converted to 1200 Hz with special ballasts (Osram Quicktronic QT-Eco 1 58/230-240), and the light from each rig was diffused by a single sheet of Rosco 216 (Germany) UV-transmitting white diffusion screen to provide an even and homogenous illumination source.

### Foraging performance

The same nine bumblebee colonies for which we had obtained laboratory colour bias data, were placed in the field (near Gieshügel, Würzburg) to measure their nectar foraging performance between 14 June and 12 July 2002. The area is typical central European bumblebee habitat, giving colonies access to multiple flower species in bloom in dry grassland, deciduous forest and farmland. A colourless Plexiglas tunnel with a system of shutters, attached to each nest entrance, allowed the observer to control the movements of bees into and out of the colony. The observer monitored the flow of forager traffic, and recorded the time and mass of each individual forager when it departed, and returned to, the nest from each foraging bout. Body mass was measured by capturing bees at the entrance of the Plexiglas tunnel as they departed and arrived and transferring them to an electronic balance (Ohaus Navigator N20330, Ohaus Corporation, USA). Departure time was when the bee was released from the vial after weighing, and arrival time was recorded when the bee first reappeared at the tunnel. We determined the foraging rate of individual workers by dividing the difference in body mass (i.e. return minus outgoing mass) by the duration of the foraging trip [21], [24], [25]. The departure mass of a bumblebee is a good estimate of her empty body mass because foragers take only very small amounts of nectar with them when leaving their colony [26], enough to fuel only a few minutes of flight [7]. Therefore the bee's mass on its return represents a true reflection of the net amount of nectar she has collected in her time away from the colony. As such, a time-adjusted difference in body mass represents a sound measure of foraging rate.

Foraging data were collected over 15 days (14, 16–18, 20–21, 26, 28–29 June, 5 and 8–12 July 2002). The number of colonies for which foraging performance could be measured simultaneously on a given day was determined by the number of observers available: on the first day (14 June) all colonies were monitored simultaneously, while on subsequent days (n = 14 days) colonies to be monitored were picked at random to match the available number of observers (n = 1–7 observers/day). Foraging data were collected for each colony during 5–10 days, producing 17–37.5 hours of continuous foraging behaviour per colony (Table 1). In order to exclude orientation and defecation flights, previous studies have considered only trips lasting at least 5 [21] or 10 [24], [25] minutes as foraging bouts. In our data, foraging bouts resulting in negative foraging rates varied considerably in length (range 3–338, median = 52 minutes; n = 59), and made up only 5 of the 9 (56%) bouts shorter than 10 minutes. Therefore we consider all bouts (n = 537; Table 1) as potential foraging bouts in subsequent analyses.

**Table 1:** Sampling effort and sample sizes for field foraging performance tests.

colony	foragers	bouts	foraging observations	
			days	duration (hh:mm)
A	26	38	5	18:04
B	33	45	9	36:11
C	30	93	10	36:31
D	30	71	9	37:34
E	23	52	3	16:39
F	32	65	7	29:15
G	23	51	6	23:57
H	41	81	6	27:50
I	17	41	10	29:05
total	255	537	65	255:06

Data presented indicate the number of individual foragers and completed foraging bouts recorded for each of the 9 colonies (A–I). The last two columns indicate the number of days on which foraging performance was assessed, and the total duration of completed foraging bouts recorded (hh:mm) per colony.

**Figure 3:**
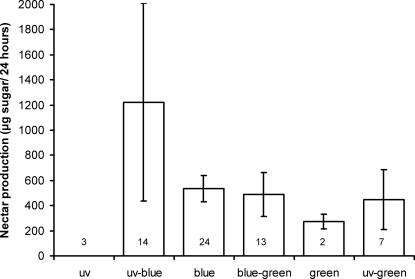
Nectar sugar production rates for plant species flowering, near Würzburg, during the period of bumblebee foraging performance experiments. The 63 plant species included all flower during this period (June and/or July) according to published phenological data [29], and are divided into six bee-subjective colour categories (numbers associated with each column indicate the number of species flowering in each colour category). Flowers were protected from visitation with gauze for 3 hours after being emptied by a *B. terrestris* worker [17], [27]. After the 3 hour exclusion period, the nectar volume was quantified for 30–60 flowers per species: nectar concentration was measured with a pocket refractometer (Atago HSR-500, Atago Co. Ltd., Japan). Data given here are the mean (± 1 SE) amounts of sugar produced (µg in 24 hours) by species in each bee-subjective colour category.

Nectar production of the flower species visited by *B. terrestris* workers near Würzburg were recorded in the spring and summer months between 1999 and 2002 [17], [27]. Production rates were calculated for each flower species from the volume and concentration of nectar produced by 30–60 flowers per species over a 3 hour period during which visitation was prevented. From these data the average amount of sugar produced (µg in 24 hours) could be calculated for each flower species. The spectral reflectance functions of all flower species were quantified, converted into bee colour space loci and placed into one of six bee-subjective colour categories: blue, blue-green, green, ultraviolet, UV-blue or UV-green [28]. To establish the nectar rewards available to our 9 test bumblebee colonies during our foraging experiment we determined which of the 75 flower species, observed to be visited by *B. terrestris* [27], were flowering during June and/ or July according to published phenology data [29]. The nectar production rates for these 63 species indicate that violet (bee UV-blue) flowers were considerably more rewarding than all other flower colours during our foraging experiments (Table 2, Fig. 3). Indeed, violet (bee UV-blue) flowers were on average more than twice (2.3×) as rewarding as blue (bee blue) flowers (the next most rewarding flower colour).

**Table 2:** Nectar sugar production rates for plant species flowering, near Würzburg, during the period of bumblebee colony foraging performance experiments.

flower species	bee-subjective colour	nectar production (µg/24 hours)
*Papaver dubium* L.	uv	0
*Papaver rhoeas* L.	uv	0
*Papaver somniferum* L.	uv	0
*Anagallis arvensis* L.	uv-b	0
*Campanula glomerata* L.	uv-b	156
*Campanula patula* L.	uv-b	75
*Cynoglossum officinale* L.	uv-b	1358
*Dactylorhiza majalis* (Rchb.) P.F.Hunt & Summerh.	uv-b	0
*Echium vulgare* L.	uv-b	1537
*Impatiens glandulifera* Royle	uv-b	11312
*Lunaria rediviva* L.	uv-b	97
*Lupinus polyphyllus* Lindl.	uv-b	0
*Lythrum salicaria* L.	uv-b	794
*Onobrychis viciifolia* Scop.	uv-b	58
*Salvia pratensis* L.	uv-b	466
*Salvia verticillata* L.	uv-b	520
*Vinca minor* L.	uv-b	731
*Allium schoenoprasum* L.	b	505
*Calluna vulgaris* (L.) Hull	b	202
*Epilobium angustifolium* L.	b	2332
*Epilobium hirsutum* L.	b	240
*Geranium robertianum* L.	b	811
*Glechoma hederacea* L.	b	160
*Lamium maculatum* L.	b	267
*Lamium purpureum* L.	b	114
*Lychnis flos-cuculi* L.	b	529
*Medicago sativa* L.	b	408
*Pinguicula vulgaris* L.	b	0
*Prunella vulgaris* L.	b	332
*Salvia nemorosa* L.	b	318
*Silene dioica* (L.) Clairv.	b	714
*Stachys palustris* L.	b	1384
*Stachys sylvatica* L.	b	898
*Symphytum officinale* L.	b	1061
*Syringa vulgaris* L.	b	500
*Teucrium chamaedrys* L.	b	221
*Thymus pulegioides* L.	b	87
*Trifolium pratense* L.	b	400
*Vicia cracca* L.	b	723
*Vinca minor* L.	b	546
*Viola canina* L.	b	97
*Cardamine pratensis* L.	b-g	745
*Centaurea jacea* L.	b-g	187
*Filipendula ulmaria* (L.) Maxim.	b-g	0
*Knautia arvensis* (L.) Coult.	b-g	141
*Lamium album* L.	b-g	467
*Linaria vulgaris* Mill.	b-g	1736
*Lunaria rediviva* L.	b-g	75
*Rosa canina* L.	b-g	0
*Sambucus nigra* L.	b-g	0
*Silene album* (Mill.) E.H.L.Krause	b-g	933
*Symphytum officinale* L.	b-g	1765
*Trifolium hybridum* L.	b-g	187
*Trifolium repens* L.	b-g	129
*Lathyrus pratensis* L.	g	216
*Lotus corniculatus* L.	g	330
*Agrimonia eupatoria* L.	uv-g	0
*Brassica napus* L.	uv-g	362
*Helianthemum nummularium* (L.) Mill.	uv-g	0
*Lamium galeobdolon* (L.) L.	uv-g	1360
*Melilotus officinalis* (L.) Pall.	uv-g	88
*Sinapis arvensis* L.	uv-g	1324
*Verbascum densiflorum* Bertol.	uv-g	0

The 63 plant species included all flower during this period (June and/or July) according to published phenological data [29], and are divided into six bee-subjective colour categories (uv = ultraviolet, uv-b = uv-blue, b = blue, b-g = blue-green, g = green and uv-g = uv-green). Data presented are mean amounts of sugar produced (µg in 24 hours) per species, averaged across at least 30 flowers per species [17], [27].

## Results

**Figure 4:**
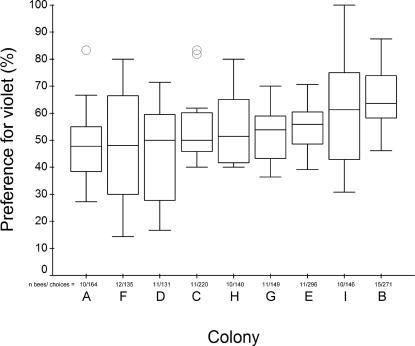
Variation among nine bumblebee colonies in their colour bias for violet (over blue) artificial flowers. In each box the thick horizontal bar is the colony median, whilst the lower and upper edges represent the 25% and 75% quartiles respectively. Whiskers indicate the maximum and minimum values that are not extreme, and outliers are represented by open circles. Outliers are data points that exceed the distance from the interquartile range box by between 1.5 and 3 times the interquartile range (SPSS Statistical software, SPSS Inc., Chicago, USA). None of these data points were excluded in any analyses. The number of bees tested and flower choices recorded for each colony are displayed along the x-axis, and colonies (A–I) are ranked by increasing colony median value from left to right.

In this bumblebee population, we observed a significant overall bias towards choosing violet (bee UV-blue) over blue (bee blue) flowers in laboratory preference tests (χ^2^ = 15.8, df = 8, p = 0.044). In the nine colonies tested, the median bias for violet (over blue) ranged from 47.7% to 63.6% (Fig. 4), with significant variation in violet bias between the colonies at either end of this range (Mann-Whitney U = 29.5, p = 0.012). In our subsequent field experiments we observed large amounts of variation in nectar foraging success, ranging from losses of 160 mg to gains of 1400 mg/ hour resulting from single foraging bouts (median = 30 mg/hour). Foraging performance across the nine test colonies differed by a factor of 1.8, from colony median foraging rates of 22 to 39 mg/hour respectively (Table 3), with significant differences in performance between the worst and best colonies (Mann-Whitney U = 1058, p = 0.006).

**Table 3:** Variation in nectar foraging performance for nine bumblebee colonies.

colony	foraging rate (mg/hr)			
	median	interquartile range	range (min/max)	bouts
A	22.02	24.82	−7.8/129.5	38
B	27.14	71.17	−112.5/255	45
C	27.93	47.70	−160/1400	93
D	25.56	53.24	−120/480	71
E	35.72	55.63	−91.6/234.2	52
F	30.26	41.34	−47.6/181.4	65
G	25.93	38.17	−70.3/372	51
H	38.82	61.25	−45.8/341.5	81
I	34.47	43.85	−24.6/300	41

Data presented are the median, interquartile range, and range (minimum and maximum) foraging rates for each colony calculated for the number of foraging bouts indicated in the last column.

**Figure 5:**
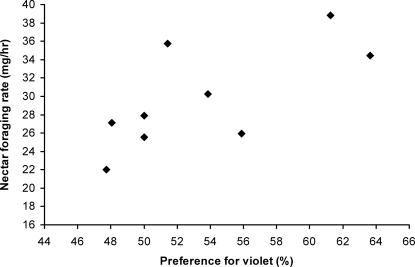
Correlation of innate floral colour preference for violet (over blue) and foraging performance in the wild, measured for nine bumblebee colonies (*B. terrestris*) near Würzburg (rs = 0.678; n = 9; p = 0.045). Each data point represents the colony median value for each of these traits.

Most importantly, we found that colonies with a stronger innate preference for violet in the laboratory harvested more nectar per unit time under natural conditions in the field (Fig. 5). Colony median nectar foraging rate was significantly correlated with colony median bias for violet (over blue: r_s_ = 0.678; n = 9; p = 0.045) for the nine colonies tested. Our results demonstrate a positive correlation between the sensory bias of *B. terrestris* colonies for violet (over blue) flowers with their nectar foraging performance under the natural conditions to which they should be locally adapted. The strength of this correlation indicates that bees from the colony with the strongest bias for violet brought in almost 41% more nectar than bees from the colony with the least strong bias. As violet flowers were on average more rewarding than blue flowers (the next most rewarding flower colour) in the local area (Fig. 3, [17]), this correlation supports our hypothesis that colonies biased towards the more highly rewarding violet flowers do collect more nectar per unit time.

This pattern is confirmed when considering these results in conjunction with those from an earlier (2001), smaller scale study with only 5 bumblebee (*B. terrestris*) colonies at the same location [9]. The results of this study also indicated a positive, though not statistically significant, correlation between colony violet preference and nectar foraging rate (r_s_ = 0.82; n = 5; p = 0.089). However, combining the results of these two independent studies produces a statistically significant result (Fisher's [30] test to combine probabilities from independent tests of significance: χ^2^ = 11.04, df = 4, p = 0.026) demonstrated for two consecutive years.

In other studies, worker body size has been shown to have a strong effect on foraging performance, with larger bees collecting proportionately more nectar [24], [25]. Although the size of workers differed significantly among colonies in our study (χ^2^ = 22.18, df = 8, p = 0.005), we found no correlation between colony median forager mass and either the strength of violet preference (r_s_ = 0.208; n = 9; p = 0.574) or foraging performance (r_s_ = 0.183; n = 9; p = 0.637) among the nine colonies. Thus colonies with larger workers did not show a stronger preference for violet (over blue flowers) or collect more nectar in our study.

## Discussion

Linking intercolony variation in sensory bias revealed under controlled laboratory conditions with the foraging performance of the same bee colonies under natural conditions represents a novel way to study the adaptive value of a foraging-related trait. Our study indicates a positive correlation between the innate preference of *B. terrestris* colonies for violet (over blue) flowers with their nectar foraging performance under the real conditions in which they operate (Fig. 5). As violet flowers were much more productive than blue flowers in the local area (Fig. 3, [17]), our findings are consistent with the hypothesis that colonies biased towards the more highly rewarding violet flowers collect nectar at a higher rate.

However, as correlation does not necessarily indicate a causal relationship, we must consider alternative explanations for the observed pattern. Potentially a spurious correlation could be produced between colony colour preference and foraging performance, if both these factors were correlated with a third variable. Body size could be one such variable because previous studies indicate that larger bumblebees are both more effective nectar foragers [24], [25] and have more sensitive eyes with greater visual acuity [31]. However, although we found significant variation in worker body size across the nine test colonies, we observed no correlation between body size and either colour preference or foraging performance in this study. Parasitism represents another potential factor which could affect our correlation, as the foraging behaviour of bumblebees can be strongly affected by parasites [32]–[34]. However, there is no evidence to suggest that parasites affect bee colour preferences, and the degree of colour preference variation observed among colonies in this study is very similar to that shown for other laboratory colonies known to be parasite free [14]. Taken together with the fact that the colour preferences of *B. terrestris* colonies are heritable [22], this suggests it is very unlikely that the variation in colour preference observed among the nine test colonies is not genetically determined.

It is easy to imagine how strong innate colour preferences help guide naïve bees to find flowers on their first foraging trip away from the nest, as very few objects except flowers fall within the blue-violet colour range in a natural landscape. Such innate colour biases presumably guide bees to investigate violet or blue objects (flowers) in preference to leaves, rocks, etc. Following the same logic, if violet flowers are consistently more rewarding than blue flowers, then it would make adaptive sense to prefer violet objects to blue ones if the bee has no other information. As bees gain foraging experience, by visiting hundreds or thousands of flowers per day [7], [35], they establish an increasingly detailed picture of which flower species (or colours) are the most profitable and when. Bees are easily able to learn to associate multiple floral traits, including colour [18], [36], morphology [37], [38], and scent [8] with levels of reward, including nectar temperature [10], and such learned associations allow individual foragers to modify, or even overwrite, their inbuilt sensory biases within a short period of time [8], [39], [40]. Despite the obvious utility to being able to modify floral choices as a result of experience in this way when foraging in a dynamic pollination market, experienced foragers also revert to their initial, innate preferences in some situations: for example when rewards are similar across a range of flower species [36], [41], [42].

Whilst such innate sensory biases appear to make adaptive sense from the bee's perspective, the question arises why flowers have not exploited these colour preferences such that violet flowers might ultimately produce less nectar than blue flowers while maintaining the same pollination success. Such a strategy would only be effective if bees always relied entirely on their innate preferences to make flower choices, which is not the case. Like other animals [1], [6], bees rely most heavily on their innate sensory biases when they are most inexperienced, i.e. during the first few foraging bouts after leaving the nest. Their level of reliance on such biases diminishes as they build up individual experience of the rewards provided by individual flower species [7], [8], [14], [36]. Hence, the vast majority of floral choices made during a bee's foraging career are at least partially informed by individual experience. Therefore overall flower visitation rates are largely dominated by the informed choices of experienced bees, rather than as a result of the sensory biases of naïve foragers.

As the colonies in our study with the strongest bias for violet flowers also foraged most effectively in the local environment, why has directional selection not eliminated intercolony variation in this foraging-related trait? Although violet flowers are on average the most productive in the local area (Fig. 3, [17]), they might not always be the most profitable. The relative profitability of a flower species, or colour, depends not only on reward production rates, but also on a variety of other factors including the activity of other flower visitors [43], [44]. In fact a uniformly strong bias in all *B. terrestris* colonies towards violet flowers, which would cause them all to seek out violet flowers, could actually drive down the average nectar reward received by each bee per violet flower visit below that for other floral colours by resource competition. Under such conditions, bees visiting other flower colours would receive more reward per visit, meaning that naïve and inexperienced foragers from colonies with a weaker bias towards violet would actually be at an advantage competing for nectar. In this way intercolony variation in such sensory biases could be operating under frequency dependent selection.

The appreciable variation in colour bias observed among colonies in this wild bumblebee population (and even among individual bees within the same colony) is in marked contrast to the limited variation in the maximum wavelength sensitivity (λ_max_) of bee photoreceptor types [16]. It appears that sensory (colour) biases are considerably more plastic evolutionary traits, presumably because tuning spectral sensitivity of photopigments is more difficult on an evolutionary scale than changing the synaptic weights that control colour preference.

Earlier studies correlating colour bias variation among bumblebee species [22], or among populations within a single bumblebee species [16], [17], [45], with differences in their respective foraging environments have provided valuable insights into patterns of bee colour bias evolution within a phylogenetic framework. Changing our emphasis and focusing on the potential adaptive significance of colour preference at the intercolony scale in this study, we add the missing link, i.e. how variation in colour biases actually affects foraging performance. Quantifying the level of local intercolony variation in a foraging-related trait (violet-blue bias) and assessing its potential effect on foraging performance using the same set of colonies, we provide a more direct test of the potential adaptive value of this sensory bias. This approach, linking demonstrations of trait variation in the laboratory with its effect on animals operating in their natural environment, represents a valuable tool which could be usefully applied to studying the adaptive value of many other foraging-related traits in future.

## References

[1] Basolo A, Endler JA (1995). Sensory biases and the evolution of sensory systems.. Trends Ecol Evol.

[2] Endler JA, Basolo A (1998). Sensory ecology, receiver biases and sexual selection.. Trends Ecol Evol.

[3] Dawkins MS, Guilford TC (1996). Sensory bias and the adaptiveness of female choice.. Am Nat.

[4] Collins SA (1999). Is female preference for male repertoires due to sensory bias?. Proc R Soc Lond B.

[5] Bruce M, Herberstein M, Elgar M (2001). Signalling conflict between prey and predator attraction.. J Evol Biol.

[6] Smith C, Barber I, Wootton RJ, Chittka L (2004). A receiver bias in the origin of three-spined stickleback mate choice.. Proc R Soc Lond B.

[7] Heinrich B (1979). Bumblebee Economics.

[8] Menzel R, Hölldobler B, Lindauer M (1985). Learning in honey bees in an ecological and behavioral context.. Experimental Behavioral Ecology.

[9] Raine NE, Ings TC, Dornhaus A, Saleh N, Chittka L (2006). Adaptation, genetic drift, pleiotropy, and history in the evolution of bee foraging behavior.. Adv Stud Behav.

[10] Dyer AG, Whitney HM, Arnold SEJ, Glover BJ, Chittka L (2006). Bees associate warmth with floral colour.. Nature.

[11] Lunau K, Maier EJ (1995). Innate color preferences of flower visitors.. J Comp Physiol A.

[12] Chittka L, Raine NE (2006). Recognition of flowers by pollinators.. Curr Opin Plant Biol.

[13] Lunau K, Wacht S, Chittka L (1996). Colour choices of naive bumble bees and their implications for colour perception.. J Comp Physiol A.

[14] Chittka L, Spaethe J, Schmidt A, Hickelsberger A, Chittka L, Thomson JD (2001). Adaptation, constraint, and chance in the evolution of flower color and pollination color vision.. Cognitive Ecology of Pollination.

[15] Çakmak I, Wells H (1995). Honey bee forager constancy: innate or learned?. Bee Sci.

[16] Briscoe AD, Chittka L (2001). The evolution of color vision in insects.. Annu Rev Entomol.

[17] Chittka L, Ings TC, Raine NE (2004). Chance and adaptation in the evolution of island bumblebee behaviour.. Popul Ecol.

[18] Giurfa M, Nunez J, Chittka L, Menzel R (1995). Colour preferences of flower-naive honeybees.. J Comp Physiol A.

[19] Pelletier L, McNeil JN (2003). The effect of food supplementation on reproductive success in bumblebee field colonies.. Oikos.

[20] Schmid-Hempel R, Schmid-Hempel P (1998). Colony performance and immunocompetence of a social insect, *Bombus terrestris*, in poor and variable environments.. Funct Ecol.

[21] Ings TC, Ward NL, Chittka L (2006). Can commercially imported bumblebees out-compete their native conspecifics?. J Appl Ecol.

[22] Chittka L, Wells H, Prete F (2004). Color vision in bees: mechanisms, ecology and evolution.. Complex Worlds from Simpler Nervous Systems.

[23] Chittka L (1996). Optimal sets of color receptors and color opponent systems for coding of natural objects in insect vision.. J Theor Biol.

[24] Ings TC, Schikora J, Chittka L (2005). Bumblebees, humble pollinators or assiduous invaders? A population comparison of foraging performance in *Bombus terrestris*.. Oecologia.

[25] Spaethe J, Weidenmüller A (2002). Size variation and foraging rate in bumblebees (*Bombus terrestris*).. Insect Soc.

[26] Allen T, Cameron S, McGinley R, Heinrich B (1978). The role of workers and new queens in the ergonomics of a bumblebee colony (Hymenoptera: Apoidea).. J Kans Entomol Soc.

[27] Raine NE, Chittka L (2007). Nectar production rates of 75 bumblebee-visited flower species in a German flora (Hymenoptera: Apidae: *Bombus terrestris*).. Entomol Gen.

[28] Chittka L, Kevan PG, Dafni A, Kevan PG, Husband BC (2005). Flower colour as advertisement.. Practical Pollination Biology.

[29] Rauh W, Senghas K, Fitschen S (1987). Flora von Deutschland und seinen angrenzenden Gebieten.

[30] Fisher RA (1954). Statistical Methods for Research Workers.

[31] Spaethe J, Chittka L (2003). Interindividual variation of eye optics and single object resolution in bumblebees.. J Exp Biol.

[32] Schmid-Hempel R, Müller CB (1991). Do parasitized bumblebees forage for their colony?. Anim Behav.

[33] Schmid-Hempel P, Stauffer H-P (1998). Parasites and flower choice of bumblebees.. Anim Behav.

[34] Schmid-Hempel P, Schmid-Hempel R (1990). Endoparasitic larvae of conopid flies alter pollination behaviour of bumblebees.. Naturwissenschaften.

[35] Raine NE, Chittka L (2007). Flower constancy and memory dynamics in bumblebees (Hymenoptera: Apidae: *Bombus*).. Entomol Gen.

[36] Gumbert A (2000). Color choices by bumble bees (*Bombus terrestris*): innate preferences and generalization after learning.. Behav Ecol Sociobiol.

[37] Laverty TM (1994). Bumble bee learning and flower morphology.. Anim Behav.

[38] Raine NE, Chittka L (2007). Pollen foraging: learning a complex motor skill by bumblebees (*Bombus terrestris*).. Naturwissenschaften.

[39] Raine NE, Ings TC, Ramos-Rodríguez O, Chittka L (2006). Intercolony variation in learning performance of a wild British bumblebee population (Hymenoptera: Apidae: *Bombus terrestris audax*).. Entomol Gen.

[40] Gegear RJ, Laverty TM (2004). Effect of a colour dimorphism on the flower constancy of honey bees and bumble bees.. Can J Zool.

[41] Heinrich B, Mudge PR, Deringis PG (1977). Laboratory analysis of flower constancy in foraging bumble bees: *Bombus ternarius* and *B. terricola*.. Behav Ecol Sociobiol.

[42] Banschbach VS (1994). Colour association influences honey bee choice between sucrose concentrations.. J Comp Physiol A.

[43] Raine NE, Chittka L (2005). Comparison of flower constancy and foraging performance in three bumblebee species (Hymenoptera: Apidae: *Bombus*).. Entomol Gen.

[44] Willmer PG, Stone GN (2004). Behavioral, ecological, and physiological determinants of the activity patterns of bees.. Adv Stud Behav.

[45] Chittka L, Briscoe A, Barth FG, Schmid A (2001). Why sensory ecology needs to become more evolutionary - insect color vision as a case in point.. Ecology of Sensing.

[46] Chittka L (1992). The color hexagon - a chromaticity diagram based on photoreceptor excitations as a generalized representation of color opponency.. J Comp Physiol A.

[47] Peitsch D, Feitz A, Hertel H, de Souza J, Ventura DF (1992). The spectral input systems of hymenopteran insects and their receptor-based colour vision.. J Comp Physiol A.

[48] Dyer AG, Chittka L (2004). Biological significance of distinguishing between similar colours in spectrally variable illumination: bumblebees (*Bombus terrestris*) as a case study.. J Comp Physiol A.

[49] Dyer AG (2006). Discrimination of flower colours in natural settings by the bumblebee species *Bombus terrestris* (Hymenoptera: Apidae).. Entomol Gen.

